# Innovations in the management of epistaxis secondary to hereditary hemorrhagic telangiectasia: our evolution to injection sclerotherapy as the treatment of choice

**DOI:** 10.3389/falgy.2024.1456686

**Published:** 2024-08-28

**Authors:** Nitish Kumar, Pedro Lança Gomes, Michael J. Marino, Amar Miglani, Devyani Lal

**Affiliations:** Department of Otorhinolaryngology-Head & Neck Surgery, Mayo Clinic, AZ, United States

**Keywords:** 3% sodium tetradecyl sulfate, sclerotherapy, epistaxis, hereditary hemorrhagic telangiectasia, genetic disorder

## Abstract

**Introduction:**

We compared the efficacy of intralesional sclerotherapy using 3% sodium tetradecyl sulfate with non-sclerotherapy-based treatments for Hereditary Hemorrhagic Telangiectasia-associated epistaxis management.

**Methodology:**

This is a retrospective study of patients who underwent surgical intervention for HHT-associated epistaxis management from 01/2010–02/2024. Patients undergoing sclerotherapy with intralesional 3% sodium tetradecyl sulfate were included in the sclerotherapy group and others undergoing conventional non-sclerotherapy-based procedures in the non-sclerotherapy group. Outcomes like breakthrough epistaxis, emergency visits, intra-op blood loss, blood transfusions, and procedure complications in the 3-month perioperative period were compared.

**Results:**

Twenty-three patients who underwent 74 intranasal procedures were identified. In the sclerotherapy group, 17 patients underwent 47 procedures. In the non-sclerotherapy group, 10 patients underwent 27 procedures. Till the 3rd post-treatment month, fewer breakthrough epistaxis episodes were observed after sclerotherapy procedures (13/47) vs. non-sclerotherapy procedures (14/27); (*p* = 0.037). Intraoperative blood loss was significantly lower during sclerotherapy (median: 10 ml) vs. non-sclerotherapy procedures (median: 50 ml); *p* < 0.001. The time interval between successive procedures was not significantly different in the sclerotherapy (median 6.5 months) vs. the non-sclerotherapy group (median 3.5 months); *p* = 0.13. Nasal crusting was the most common complication in the sclerotherapy group (36.9%). Two patients in each group had new onset septal perforation, none of the patients had vision loss or cerebrovascular accident. One emergency department visit was reported in the sclerotherapy group vs. 7 (in 3 patients) in the non-sclerotherapy group.

**Conclusions:**

Compared to non-sclerotherapy treatments, intralesional sclerotherapy for epistaxis in HHT is more effective in decreasing breakthrough epistaxis, and has lower intraoperative blood loss.

## 1 Introduction

Hereditary hemorrhagic telangiectasia (HHT) is an autosomal dominant disorder with variable penetrance that leads to progressive loss of elastin from the blood vessel walls (1). This leads to arteriolar and capillary dilation, enhanced microvascular flow, and eventual disappearance of the capillary bed giving rise to direct arterial to venous blood flow. As vessels enlarge, they become tortuous and lie closer to the mucosal surface, with an enhanced risk of exposure and trauma. These vessels give rise to recurrent epistaxis episodes which are difficult to control as vasoconstriction is hampered due to loss of elastin (2). Such epistaxis episodes of variable severities can lead to complications ranging from anemia to severe hemorrhage mandating an emergency department (ED) visit.

Repeated interventions are required to control this recurrent epistaxis. Current surgical techniques include endoscopic cauterization of the bleeder, debridement of the telangiectasias, endoscopic coblation of the bleeder, laser photocoagulation, endoscopic embolization of facial and internal maxillary artery, intralesional bevacizumab injections, along with older techniques for dealing with severe epistaxis like nasal closure (Young's procedure), and septodermoplasty. All these procedures have reported variable efficacy for epistaxis control and are associated with complications like nasal crusting, foul smell, and nasal septal perforation (3). Repeated need for these procedures further enhances the risks of these complications. Both patients and their treating otolaryngologists still struggle to achieve effective HHT-associated epistaxis (HHT-Ep) relief. A procedure that is effective for epistaxis control, simple to perform, safe, and offers a relatively longer period of epistaxis control is desirable in these patients. Sclerotherapy with intralesional injection of 3% sodium tetradecyl sulfate (STS) into individual telangiectasias has been reported to be efficacious in controlling HHT-Ep (1, 4, 5). Initial studies have also demonstrated sclerotherapy to be a relatively safe procedure with the potential to provide an office-based treatment (1, 4). First study reporting use of sclerotherapy dates back to 2011, using 3% STS (1). In the same year, Morais, et al. (6) reported their positive experience with use of 1% polidocanol injection for HHT-Ep management. This was followed by studies reporting complications of 3% STS (7) and its efficacy compared to conventional procedures (4, 5), and a single study reporting efficacy of 1% polidocanol for HHT-Ep management in 2021 (8). However, wider adoption of this technique has been hampered due to concerns of blindness secondary to accidental injection into the ophthalmic circulation, which has only been reported with fibrin glue injection (9). Other possible complications of sclerotherapy include pain, nasal crusting, nasal discharge, and septal perforation, all of which precipitate due to extravasation of sclerosant during injection leading to localized tissue cellulitis. Hence, careful and low volume injection with gradual withdrawal is important to minimize extravasation (7).

We present our early experience with intralesional sclerotherapy using 3% STS, comparing its outcomes with non-sclerotherapy-based treatments for HHT-Ep.

## 2 Methodology

This study was approved by the Mayo Clinic Institutional Review Board (IRB no. 24-000751). Patients who underwent surgical intervention for HHT-Ep management from 01/2010–02/2024 at Mayo Clinic, Arizona were selected for a retrospective review of electronic medical records. Patients receiving sclerotherapy-based management with 3% intralesional sodium tetradecyl sulfate (STS) were included in the sclerotherapy group and others receiving non-sclerotherapy-based interventions like cauterization, coblation, microdebrider-assisted removal of telangiectasias, etc. were included in the non-sclerotherapy group. The 3 months immediately preceding and following the intervention were studied to record the following data: patient demographics (age, sex), significant epistaxis episodes (epistaxis episodes not self-limited and lasting >1 h), intraoperative blood loss, emergency visits needed for epistaxis management, blood transfusions needed due to an acute severe epistaxis episode or chronic anemia due to repeated epistaxis, surgical complications (nasal crusting, nasal septal perforation, acute vision loss, and neurological deficits), and time between successive procedures. Standard method of 3% STS-based sclerotherapy included preparation of sclerosant in 1:4 ratio with air using the double syringe system. Under general anesthesia, telangiectasias were visualized and injected with the foamed sclerosant. The injection was a slow, low- volume low-pressure injection, continued until the blanching of lesion. This was followed by a brief pause and gradual withdrawal of needle to ensure simultaneous coagulation of the vessel and prevent extravasation. Maximum volume of foamed sclerosant to be used was capped at 3 ml to prevent pushing it into the ophthalmic circulation and avoid the catastrophic complication of blindness.

Microsoft Excel and STATA BE/18.0 were used for statistical analysis. Pearson's Chi-square analysis was used to assess the difference in breakthrough bleeding. Mann-Whitney U test was used to assess differences in the time interval between successive procedures and intraoperative blood loss between the 2 groups. A *p*-value of <0.05 was chosen as a criterion of statistical significance with a 95% confidence interval.

## 3 Results

Seventy-four episodes of surgical intervention for HHT-Ep in 23 patients were identified. The mean age of patients (±1 standard deviation) in the sclerotherapy group was 62.1 (±11.1) years and in the non-sclerotherapy group was 75.7 (±8.25 years). Four patients underwent non-sclerotherapy-based interventions at our center before switching to sclerotherapy, hence, they were common in both groups. Before presenting to us, 19 out of 23 patients (13/17 patients in the sclerotherapy and 10/10 patients in the non-sclerotherapy group) had undergone prior non-sclerotherapy-based procedures like laser photocoagulation, intranasal bevacizumab injection ± cauterization ± microdebrider assisted removal of telangiectasias, coblation, young's procedure, and septodermoplasty) for HHT-Ep management at other centers.

Seventeen patients underwent sclerotherapy with 3% STS and 10 underwent non-sclerotherapy-based interventions namely laser photocoagulation, intranasal bevacizumab injection ± cauterization ± microdebrider assisted removal of telangiectasias, coblation, and embolization of facial and internal maxillary artery. All patients experienced significant episodes of epistaxis during the preoperative period. Details of procedures performed in the non-sclerotherapy group are given in Table 1.

**Table 1:** Details of procedures in the non-sclerotherapy group.

Name of the procedure	No. of times performed
Endoscopic electrocauterization	6
Endoscopic electrocauterization with topical bevacizumab injection	9
Endoscopic electrocauterization with debridement of telangiectasias	6
Endoscopic electrocauterization with debridement of telangiectasias with topical bevacizumab injection	2
Topical bevacizumab injection	1
Endoscopic coblation	2
Embolization of internal maxillary artery and facial artery	1

The non-sclerotherapy group experienced significantly more episodes of breakthrough epistaxis [14/27 (51.8%)] compared to those who underwent sclerotherapy with 3% STS [13/47 (27.6%)]; *p* = 0.037. Intraoperative blood loss was significantly lower in the sclerotherapy group with a median of 10 ml [inter-quartile range (IQR): 5–30 ml] compared to the non-sclerotherapy group with a median blood loss of 50 ml (IQR: 25–150 ml); *p* < 0.001. Patients experienced longer remission periods from epistaxis in the sclerotherapy group, with a median of 6.5 months (IQR: 3–10 months) in the sclerotherapy vs. a median of 3.5 months (IQR: 1.5–6 months) in the non-sclerotherapy group, however, this difference did not reach statistical significance (*p* = 0.13). The most frequent complication of sclerotherapy was crusting (36.9%) followed by nasal discharge (10.8%). In the non-sclerotherapy group, 48.14% of patients experienced nasal crusting. Two patients in each group had new onset septal perforation post-intervention and underwent successful septal perforation repair. None of the patients had any visual problems post-intervention. Post-procedure complications of both groups are listed in Table 2. No patients were packed after either sclerotherapy or non-sclerotherapy treatments. In the sclerotherapy group, 6 patients visited the emergency department (ED) 8 times for epistaxis management in the 3-month pre-operative period, which was reduced to a single visit in the 3-month post-sclerotherapy period. In the non-sclerotherapy group, 3 patients visited the ED 8 times preoperatively, and in the postoperative period, 3 patients again required 7 visits to the ED for HHT-Ep management. Four patients in the sclerotherapy group received 4 blood transfusions due to severe anemia or severe epistaxis episodes preoperatively whereas postoperatively only a single blood transfusion was required in 1 patient. In the non-sclerotherapy group, 2 patients required 5 blood transfusions preoperatively and 2 patients required 2 blood transfusions in the postoperative period. Table 2 displays demographics of the study population and the results of this study.

**Table 2:** Demographic and clinical details of cases.

	Sclerotherapy group	Non-sclerotherapy group	*p*-value
No. of patients	17	10	
Mean age (yrs.)	62.1 ± 11.1	75.7 ± 8.25	
Sex distribution (M/F)	7 (41.2%)/10 58.8%)	6 (60%)/4 (40%)	
No. of procedures	47	27	
Median time interval between successive procedures (IQR)	6.5 months (3–10)	3.5 months (1.5–6)	*p* = 0.13
Troublesome HHT-Ep episodes in the preoperative period	46 (97.9%)	27 (100%)	
Breakthrough bleeding before completion of 3rd post-op month	13 (27.6%)	14 (51.8%)	*p* = 0.037
Median intraoperative blood loss (IQR)	10 ml (5–30)	50 ml (25–150)	*p* < 0.001
Procedure complications:			
• Crusting • Nasal discharge • Nasal septal perforation • Vision loss • Neurological deficit/CVA	17 (36.9%)	13 (48.14%)	
	5 (10.8%)	0	
	2	2	
	0	0	
	0	0	
No. of pre-op emergency visits	8 (6 patients)	8 (3 patients)	
No. of post-op emergency visits	1	7 (3 patients)	
No. of pre-op blood transfusions	4 (4 patients)	5 (2 patients)	
No. of post-op blood transfusions	1	2 (2 patients)	

## 4 Discussion

Sodium tetradecyl sulfate is a detergent-type sclerosant introduced in 1946 for managing varicose veins (10). Sclerosants act by inducing endothelial damage with thrombophlebitis of the injected vessel. Foam sclerotherapy has the added advantage of providing an exponentially large area of contact between sclerosant and endothelium, thus decreasing its required volume. High viscosity minimizes washing away of the sclerosant and prolongs the duration of its contact with endothelium, thus facilitating its action. Vasospasm follows sclerosant injection which ensures approximation of the injured endothelium of the injected vessel resulting in intravascular scarring and collapse, increasing the likelihood of long-term occlusion of the vessel (11). This is desired in the patients of HHT who suffer recurrent epistaxis episodes due to exposed, dilated, and tortuous vessels prone to trauma. We used the foaming technique described by Tessari and Frullini in 1997 (11), which was also used by Boyer, et al., (1) who were the first to demonstrate the efficacy of injection sclerotherapy with 3% STS. Foaming of 3% STS was done in an air-to-sclerosant ratio of 4:1, which is deemed most stable, using the double syringe system (11).

We observed a significant reduction in breakthrough epistaxis post-intervention in the sclerotherapy group compared to the non-sclerotherapy group (*p* = 0.037). This was also reflected in the no. of ED visits required in the perioperative observation period which reduced from 8 to 1 post-sclerotherapy compared to 8 pre- and 7 post-intervention with non-sclerotherapy-based management. Previous studies identified similar outcomes with 3% STS-based sclerotherapy. Boyer, et al., (1) were the first to perform a pilot study evaluating the efficacy of 3% STS-based sclerotherapy. They noted a significant reduction in the frequency and severity of epistaxis post-intervention, assessed using a patient-reported questionnaire. They also observed a reduced need for blood transfusion post-sclerotherapy. This was followed by a prospective cross-over study between patients receiving 3% STS-based sclerotherapy and standard non-sclerotherapy-based treatments by Boyer, et al. (4) They found significantly reduced epistaxis episodes with 3% STS-based sclerotherapy assessed using the epistaxis severity score (ESS). In a larger cohort, Woodard, et al. (5), retrospectively demonstrated a reduced frequency of procedures for HHT-Ep control with 3% STS-based sclerotherapy compared to cauterization or laser photocoagulation.

We observed significantly lower (*p* < 0.001) intraoperative blood loss (median 10 ml) with sclerotherapy vs. non-sclerotherapy interventions (median 50 ml). Sclerotherapy has an immediate onset of action, and a gradual needle withdrawal provides ample time for simultaneous coagulation of the target vessel (4). This reduces the likelihood of intraoperative hemorrhage and the need for nasal packing. None of the patients in our study needed nasal packing in the immediate postoperative period. This favors a possible office administration of sclerotherapy for select HHT patients. Boyer, et al., (1) reported in the pilot study that no patient had intraoperative bleeding requiring intervention.

We also observed a relatively longer interval between successive sclerotherapy procedures (median 6.5 months) compared to the non-sclerotherapy group (median 3.5 months), although it was not found to be a statistically significant difference (*p* = 0.13). This indicates that fewer sclerotherapy procedures might be needed for long-term HHT-Ep control. In a recent study, Woodard, et al., (5) observed a significantly lesser requirement of 3% STS-based sclerotherapy procedures by HHT patients to keep ESS in the mild range, compared to laser photocoagulation and electrocauterization during a 24-month observation period. The combined thrombophlebitic and vasospastic effect of foam sclerosant leading to permanent collapse of the target vessel might be responsible for the long duration of remission, although, this observation remains unexplored. The effect of sclerosants on new telangiectasia development is also not known. On the other hand, a standard procedure like electrocauterization is associated with an accelerated rate of new telangiectasia formation along with higher surrounding tissue damage (8).

Complications of 3% STS-based sclerotherapy noted in our study patients included mainly nasal crusting (36.9%) and discharge (10.8%). These are the aftermath of extravasation of the sclerosant in the surrounding tissue leading to inflammation and tissue necrosis. Also, the dreaded complication of injection sclerotherapy for HHT-Ep is the risk of accidental injection in the ophthalmic circulation which can lead to complete loss of vision. This has been reported with the use of fibrin glue (9). A precisely located, low-pressure, low-volume sclerosant injection can avoid this complication and reduce extravasation avoiding nasal crusting, discharge, and pain (1). It is recommended that the volume of 3% STS for a single sclerotherapy procedure should not exceed 3 ml (1). In our study, none of the patients in either group had a complication of vision loss/reduced visual acuity or cerebrovascular accident. None of the studies in the published literature report vision loss or reduced visual acuity as a complication of 3% STS-based sclerotherapy for HHT-Ep (1, 4, 5, 7). Another concerning complication is septal perforation, which was observed in 2 patients in both groups. Bilateral septal injections across the same site were likely responsible for the septal perforations in the sclerotherapy group and hence, should be avoided (6). Septal perforation risk increases if patients have had prior septoplasty or prior interventions for epistaxis management (23 of 27 patients), therefore we avoid septoplasty in our HHT patients. We also recommend repairing nasal septal perforations in HHT patients as they can further worsen the ongoing recurrent epistaxis. Both patients in the sclerotherapy group underwent successful septal perforation repair at our center.

An interesting aspect to observe in future studies will be use of sclerotherapy combined with conventional procedures. Although it is not mentioned in the published literature, in our experience it is challenging to address far posteriorly located telangiectasias with intralesional sclerotherapy. Use of coblation or electrocautery for such lesions could further improve HHT-Ep control. How the choice of existing conventional procedures, in combination with 3% STS based sclerotherapy, affects the outcomes of HHT-Ep control will be interesting to observe. Another interesting observation will be the variation in control of telangiectasias located on different sites in the nasal cavity, namely on septum, turbinates, and rest of the lateral nasal wall. Such knowledge can further help us choose the most site-suitable procedure for HHT-Ep control.

Currently there is very limited data comparing efficacy of sclerotherapy with existing conventional procedures, to which we aim to add with our experience. This study also highlights the efficacy and safety profile of 3% STS as an agent to be used for intralesional injection sclerotherapy. This is also the first study to report the difference of intraoperative blood loss between non-sclerotherapy and sclerotherapy-based procedures, a characteristic of vital importance in HHT patients who generally are already suffering from recurrent blood loss and anemia. The design of this study (retrospective chart review) has inherent limitations for the generalization of these results. HHT is a rare disease with an incidence of 1 in 5,000 (1), hence, we had a small sample size. We included all patients who underwent non-sclerotherapy-based procedures in 1 group for comparison with the sclerotherapy group due to the small available sample size, as previously done by Boyer, et al. (4), which is not ideal. Standardized epistaxis questionnaires like ESS were not available. Assessment of epistaxis severity was based on the presence or absence of episodes that were not self-controlled and required medical attention, or lasted >1 h, as mentioned in the methodology section.

Our findings are not novel, but more centers need to publish their results on the efficacy and safety of sclerotherapy to address concerns regarding the dreaded complication of vision loss. In the last 7–8 years, multiple rhinologists at our center have shifted to intralesional sclerotherapy for managing HHT-Ep after witnessing promising outcomes and improved patient satisfaction. Careful sclerotherapy appears to be safe and offers relief from epistaxis to patients who suffer severe detriment to their quality of life and health from HHT.

## 5 Conclusions

Sclerotherapy with 3% STS is an effective alternative for HHT-associated epistaxis. Compared to other interventions, sclerotherapy is associated with minimal intraoperative blood loss and may offer superior control of breakthrough epistaxis with longer periods of epistaxis-free remission. Prudent technique with foamed STS and careful intra-telangiectasia injection increases efficacy, lowers the amount of sclerosant used, and reduces the risk of injection into the ophthalmic circulation leading to a risk of blindness. Clinicians should exercise shared decision-making with their patients to consider sclerotherapy for managing HHT-associated epistaxis after thoughtful consideration of risks vs. benefits.

## Data Availability

The raw data supporting the conclusions of this article will be made available by the authors, without undue reservation.
